# Macrophage Polarization and Plasticity in Systemic Lupus Erythematosus

**DOI:** 10.3389/fimmu.2021.734008

**Published:** 2021-12-20

**Authors:** Mariame Mohamed Ahamada, Yang Jia, Xiaochuan Wu

**Affiliations:** Department of Pediatrics, The Second Xiangya Hospital, Central South University, Changsha, China

**Keywords:** systemic lupus erythematosus, macrophage activation syndrome (MAS), M1 macrophage polarization, M2 macrophage polarization, macrophage plasticity and polarization

## Abstract

Systemic lupus erythematosus (SLE) is an autoimmune disease that attacks almost every organ. The condition mostly happens to adults but is also found in children, and the latter have the most severe manifestations. Among adults, females, especially non-Caucasian, are mostly affected. Even if the etiology of SLE remains unclear, studies show a close relation between this disease and both genetics and environment. Despite the large number of published articles about SLE, we still do not have a clear picture of its pathogenesis, and no specific drug has been found to treat this condition effectively. The implication of macrophages in SLE development is gaining ground, and studying it could answer these gaps. Indeed, both *in vivo* and *in vitro* studies increasingly report a strong link between this disease and macrophages. Hence, this review aims to explore the role of macrophages polarization and plasticity in SLE development. Understanding this role is of paramount importance because in-depth knowledge of the connection between macrophages and this systemic disease could clarify its pathogenesis and provide a foundation for macrophage-centered therapeutic approaches.

## 1 Introduction

Systemic lupus erythematosus belongs to a group of autoimmune diseases commonly called lupus. Lupus means “wolf” in Latin and describes a facial rash resembling a wolf bite (1). Initially, the disease was simply called lupus because it was thought only to involve the skin. Later, when other systemic manifestations were reported, the name Systemic lupus erythematosus (SLE) was introduced (2). So far, there is no known direct cause of this disease. However, its higher distribution in adults, female gender, and certain ethnicities such as non-Caucasian, makes genetics and environment two crucial determinants of disease development (3, 4). SLE affects almost every organ and presents with a high range of clinical manifestations (5). The damage to various organs can be explained by a disturbance of innate and adaptive immune responses leading to a production of autoantibodies, immune complexes, and a loss of immune tolerance to autoantigens (6, 7). It is well known that innate and adaptive immunity work together to defend an individual against pathogens. However, in some disorders such as SLE, this function is deregulated. Consequently, the immune system attacks the individual’s self-organs instead of protecting them. Among the immune components, macrophages are believed to play a significant role in the pathogenesis of SLE. Indeed, it has been established that the polarization of macrophages into M1-/M2-like macrophages affects the development of lupus (8). A significant influence on the disease process is also exerted by macrophage plasticity. This unique property of macrophages could enable the pursuit of these immune cells for therapeutic goals by inducing a phenotype switch between M1-/M2-like macrophages (9). Given the crucial role macrophages play in the development of SLE, studies on this autoimmune disease are increasingly focusing on its relationship with these immune cells. Therefore, the purpose of this review is to provide an up-to-date summary of the connection between Systemic lupus erythematosus and macrophages.

## 2 Macrophage Polarization and Plasticity

One of the indispensable arms of the immune system is macrophages. These large multitasking and plastic cells polarize into different phenotypes and ultimately carry different functions depending on the microenvironment. Here, polarization can be understood as the spatiotemporal activation of macrophages at a given point (10, 11). In other words, polarization is the induction of functionally distinct macrophages with regard to the dominant factors in the inflammatory zone. Based on their polarization and function, macrophages were traditionally categorized into two main phenotypes. However, this long-held view is obsolete and is nowadays considered to be an oversimplified approach. Indeed, accumulating evidence affirms the existence of other macrophage populations *in vivo* (12, 13) and reveals different behaviors between *in vitro* and *in vivo* macrophages. More interestingly, it is suggested that macrophages express different genes *in vitro* and *in vivo* (14). Therefore, it is evident that the nomenclature of macrophages is not as simple as it was thought to be, and it would be erroneous to identify them as M1/M2 macrophages.

While macrophage activation and polarization allow macrophages cells to acquire a specific phenotype, macrophage plasticity, on the other hand, enables these immune cells to switch from one phenotype to another (15). In other words, these plastic cells have the unique ability to re-polarize in response to environmental factors and adopt a new phenotype. In diseases like SLE, where deregulation of macrophage phenotypes is known to play a pathogenic role, this functional adaptability has a tremendous therapeutic value because it could be exploited to restore the balance between different macrophages subtypes. Thus, we must review the diversity of macrophage phenotypes in SLE development and the impact of the environment on the behavior of these immune cells.

## 3 Macrophage Phenotypes in SLE

**Figure 1:**
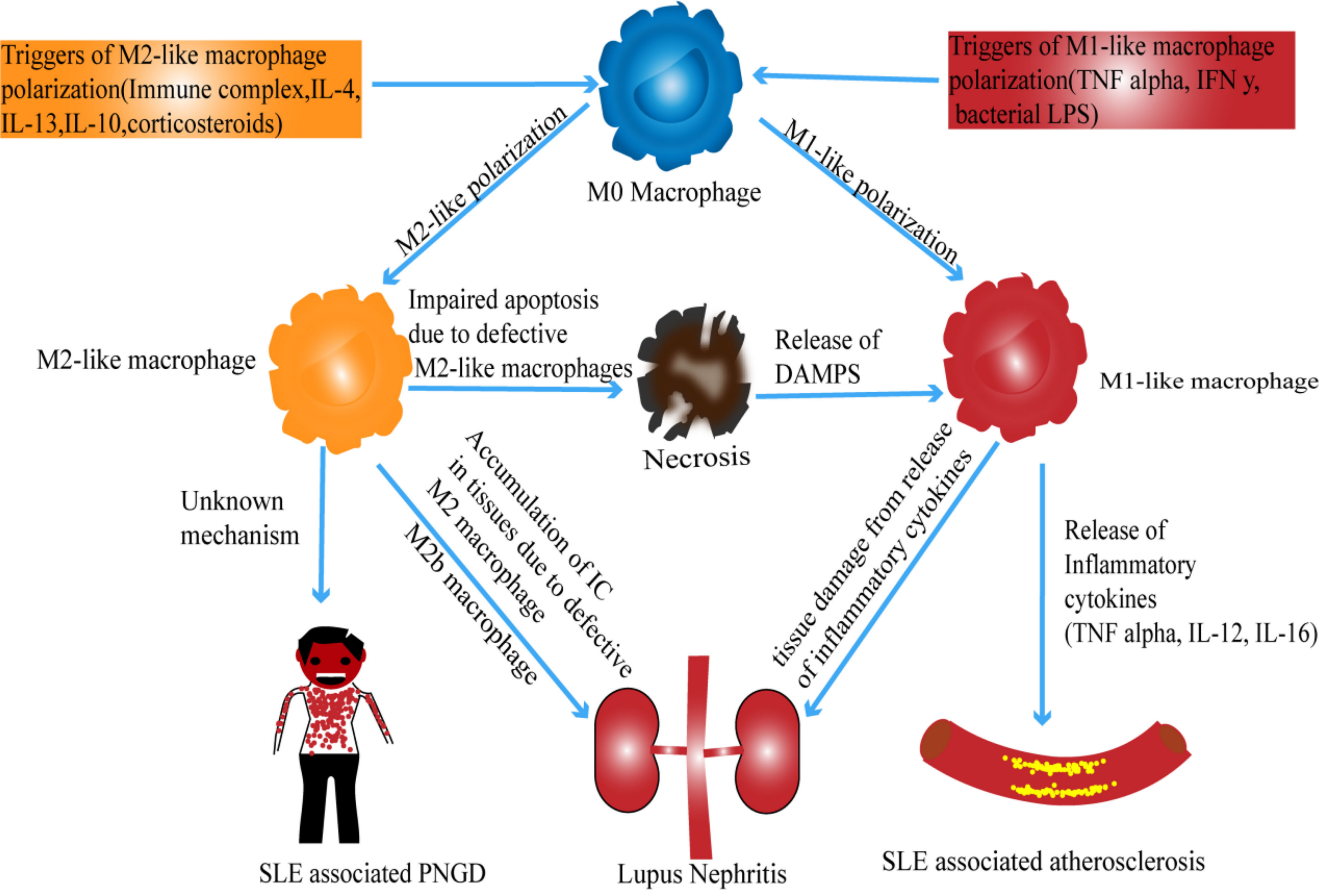
Possible mechanism of macrophage polarization in SLE.

Macrophages play an essential role in inflammatory reactions. Based on how they have been polarized, they can either exert a pro-inflammatory effect or an anti-inflammatory effect (16). Such a feature, among others, makes macrophages a potential participant in the development of inflammatory and autoimmune diseases (17). Hence, in the following sections, we explore the link between macrophage subsets and SLE development. This connection is also summarized in **Figure 1**.

### 3.1 M1-Like Macrophages in SLE

Previously, macrophages were assumed to have an M1 phenotype when their polarization was mediated by T helper one cytokines such as INF-γ. However, this hypothesis is now deemed erroneous because *in vitro* polarization can be achieved with bacterial lipopolysaccharide (LPS) without the intervention of T lymphocytes (9, 18). Functionally, these types of macrophages are known to have pro-inflammatory activity mainly. Their ability to produce inflammatory cytokines makes them, on the one hand, an essential participant in the elimination of pathogens; however, on the other hand, they interfere with wound healing and tissue repair (9, 19). More interestingly, M1-like macrophage-derived cytokines are believed to mediate autoimmune and chronic inflammatory diseases (20).

The role of M1-like inflammatory macrophages in SLE development is reported in many articles (17, 21, 22). Recently, clinical research has further demonstrated a positive correlation between the number of monocytes expressing M1 macrophage-like markers (CD163^-^CD14^+^) in peripheral blood of children with lupus and the severity of childhood-onset SLE (23). Even though CD14 and CD163 are not specific for macrophages, CD163^-^CD14^+^ cells are considered M1-like cells (24), and therefore, there is a possibility that the disease activity observed in this study is associated with M1-like macrophages. The connection between M1-like macrophages and SLE can also be appreciated in Lupus nephritis (LN). LN, one of SLE complications, is believed to be characterized by a deregulation of both M1-and M2-like macrophages. Evidence suggesting the involvement of the M1 phenotype showed that LN could be mediated *in vivo* by type I interferon signature, to which M1 macrophages are very responsive (25). This implication of M1 macrophages is additionally supported by their involvement in the development of atherosclerosis. Atherosclerosis poses a significant threat to global health, and its incidence is high in young patients with SLE. In these subjects, various immune cells, specifically macrophages, are thought to be involved in developing this vascular disease. Whereas the exact role of macrophages in the progression of SLE-associated atherosclerosis is not extensively investigated, the increased serum neopterin concentration in patients with SLE-atherosclerosis indicates a possible association of macrophages with atherogenic mediators of inflammation (26, 27). Additionally, it has been reported that M1-like macrophage-derived cytokines such as TNFα, IFN γ, IL-6, and IL-12 are pro-atherosclerotic and found in patients with SLE-induced atherosclerosis (28). Also, oxidative stress in the plaque seems to be worsened by nitrogen species and reactive oxygen. These molecules are generated from M1-like macrophages and could be seen in patients with SLE-associated atherosclerosis (28). Such information could allow researchers to find therapeutic targets that may improve atherosclerosis in SLE patients.

Likewise, factors that can promote the polarization of M1 macrophages can exacerbate inflammatory conditions such as lupus. Among these factors are Microparticles (MP), remarkably immune complex-forming microparticles (MP-IC). In an *in vitro* study, these extracellular vesicles were seen to favor the polarization of M1-like inflammatory macrophages and thus amplify inflammation and autoimmunity in diseases such as SLE (29). High-mobility-group box chromosomal protein (HMGB1) might be another facilitator of M1-like inflammatory macrophages (8, 30, 31). *In vitro* and *in vivo* studies showed that These proteins can induce SLE by promoting macrophage inflammatory response (32). In conformity with this theory, *in vitro study* found that the serum level of HMGB1 increased with the severity of neuropsychiatric SLE (NPSLE) (33). However, because evidence showed that inflammation could be mediated by other types of macrophage other than M1-like macrophages (14), we cannot confidently conclude that these inflammatory macrophages are indeed M1-like macrophages. Further research is needed to clarify this point.

These findings address the association of macrophages with SLE and point out the implication of M1-like macrophages in the pathogenesis of the disease. Nevertheless, it should be noted that non-negligible differences can be appreciated within macrophages if observed in different environments (*in vivo*/*in vitro*). For example, differences in biomarkers were found between *in vivo* and *in vitro* M1-like macrophages (14). This means *in vitro* results can not necessarily be translated into *in vivo* and vice versa. Such a concept needs to be taken into consideration when interpreting results from studies conducted in different environments. Immunological biomarkers that can help identify *in vivo* and *in vitro* M1-like macrophages are summarized in **Table 1**.

**Table 1:** *In Vivo/in Vitro* macrophage markers and their corresponding roles.

Macrophage phenotype	Immunological biomarkers	Roles
**M-1 like macrophages**		
**In Vitro M1-like macrophages**	Cxcl1, Cxcl2, Cxcl5, Ccl3, Cxcl10, Cxcl11, Ccl25, Cx3cr1, Ccr7, IL-1α, IL1β, IL-6 and TNF Traf2, Tnfaip3	Recruit neutrophils Attract T cells Inflammatory mediator (34) Immune cells migration, differentiation and activation (35) Induce pro-inflammatory cytokines
**In vivo M1-like macrophages**	Ccl2, Csf2, IFNβ1, Irf1, IL23a, IL15	Promote M1 polarization Pro-inflammatory cytokines
**In vitro and In vivo M1-like macrophage**	CD86, CD40, CD38, Cxcl16, Cxcl19 IL15ra, IL17ra	Ligand for the inflammatory marker CD28 Vascular inflammation Unclear Support M1 polarization,
**M2-like macrophages**		
**In vitro M2-like macrophages**	CD206, CD99 Bcl2, CD74, CD36	Promote anti-inflammatory cytokines Negatively regulate LPS induced activation Inhibits pro-apoptotic protein Regulate cell survival Phagocytosis and apoptic cell recognition
**In vivo M2-like macrophages**	PPARy, parp1,	Enhance CD36 expression and M2-like response Pro-inflammatory
**In vitro and In vivo M2-like macrophages**	CD84, CD300a P2Y1, GPCR	Not known, Not entirely understood Regulate inflammation and immunity
**In vivo M1 and in vitro M2-like macrophages**	Ccl7, Ccl17, Ccl22, Ccl24, CD83, CD44	Promote attraction of immune inhibitory cells
**In vitro M1- and in vivo M2-like macrophages**	Tnfrsf21 (14)	Promote apoptic processes (14)

### 3.2 M2-Like Macrophages in SLE

M2-like macrophages mainly have anti-inflammatory properties (17). However, recent studies showed that these cells might also exert a pro-inflammatory function. Indeed, poly (ADP-ribose)-polymerase1 (parp1), which represents the majority of poly (ADP-ribose)-polymerase (PARPS), do have pro-inflammatory functions even though they are present in M2-like macrophages *in vivo* (14, 36). This statement highlights the significant influence that the environment (*in vitro/in vivo*) has on the behavior of macrophages and why much attention needs to be paid when defining macrophage polarization. It is well accepted that the polarization of M2-like macrophages is induced by Th2 cytokines IL-4 and IL-13. Besides IL-4 and IL-13, other cytokines such as IL-10, IL-33, and IL-21 can also drive M2 polarization (9). Depending on the stimulating factor, M2-like macrophages can further be classified into different subtypes with distinct functions. M2a subtypes are triggered by IL-4 and IL-13 and participate in tissue repair (37). M2b macrophages are stimulated by fcyR/TLR and immune complexes and contribute to tumors and infections. As for M2c macrophages, they respond to glucocorticoid, TGF-β, and IL-10 and exert anti-inflammatory effects (38). M2d macrophages, on the other hand, represent a new subset and take part in angiogenesis and cancer metastasis (39–41). However, *in vivo* translation of these M2 subtypes is not that simple because some macrophages are found to express mixed phenotypes (42), and others do not meet the M1/M2 model (43). For this reason, the M2 classification is believed to be more about the macrophages’ activation stimuli rather than the resulting functions of these macrophages (39). **Table 1** summarizes *in vivo*/*in vitro* M2-like macrophages biomarkers.

M2-like macrophages have been reported to play a vital role in the development of SLE. Although both M1-and M2-like macrophages contribute to the pathogenesis of lupus nephritis, several studies suggest that the M2 phenotype is the dominant subpopulation (21, 44, 45). It was reported that defective M2-like macrophages could uncontrollably produce cytokines that contribute to the development of SLE (46, 47). Similarly, the inability to clear immune complexes (ICs) by defective M2-like macrophages results in organ damage by allowing ICs to accumulate in different tissues (48). Mechanically, apoptotic cells are eliminated by M2-like macrophages in a normally non-inflammatory response named efferocytosis (49). Such response gives rise to an increase of anti-inflammatory cytokines and a reduction of pro-inflammatory cytokines (50). Therefore, the inflammation in LN could be caused by nonfunctional M2-like macrophages, which have lost their anti-inflammatory property. This assumption is supported by evidence suggesting that M2-like macrophages found in LN lacked heme oxygenase-1 (HO-1) expression and that supplementation of HO-1 could ameliorate LN (51). In contrast, another research has demonstrated that Granulin (GRN), a protein linked to inflammation, can worsen LN by enhancing M2-like macrophage polarization, specifically M2b polarization (52). Although the M2b macrophages-induced LN mechanism is not clarified in this study (52), it is reported elsewhere that M2b macrophages can produce both pro-and anti-inflammatory cytokines (9, 11). Thus, further study is required to clarify whether these M2b macrophages do indeed mediate LN *via* the release of inflammatory cytokines or they are just nonfunctional macrophages that have lost their anti-inflammatory activity.

A recent study has further revealed another possible role played by M2-like macrophages in the pathogenesis of palisaded neutrophilic and granulomatous dermatitis (PNGD), a skin condition found in systemic autoimmune diseases like SLE. The study examined two cases of SLE patients with PNGD and revealed CD163+ M2- like macrophages to be the primary phenotype (53). Even if a similar hypothesis was previously deduced from evidence showing a higher expression of CD163 in SLE skin lesions (54), it is still premature to confidently speculate that M2-like macrophages are undoubtedly responsible for the pathogenesis of PNGD firstly because CD163 is not a specific marker to macrophages (55–57). Secondly, because the study was conducted on only two patients, and finally, the pathogenesis is not fully understood. Further investigations are therefore needed.

Interestingly, MicroRNAs (miRNAs), a class of small non-coding RNAs, are reported to contribute to SLE development by promoting both M1-and M2-like macrophages. Indeed, evidence showed that miRNAs are involved in SLE progression by inducing M2-like macrophages polarization through activation of lymphocyte-derived DNA (ALD-DNA) (58). Conversely, other articles reported that miRNAs induce inflammation by favoring M1 polarization (59). Besides indexing the complexities of the behavior of macrophages in diseases and health, these data also highlight why macrophages cannot simply be categorized as builders or killers. Despite the oversimplified classification of these immune cells, we cannot simply speculate that M1-like macrophages only contribute to SLE development while M2-like macrophages alleviate the disease.

## 4 Macrophage Activation Syndrome in SLE

### 4.1 Macrophage Activation Syndrome

Macrophage activation syndrome (MAS) is a rare but severe complication seen in rheumatic disorders. Although it occurs in many rheumatic diseases, it is most common in idiopathic juvenile arthritis, SLE, and Kawasaki disease (60, 61). Recent studies have further revealed that MAS is most likely in SLE when the disease is diagnosed during childhood (62, 63). And even more likely when the child presents with lupus pancreatitis (64, 65). Clinically, the syndrome is represented by hepatic dysfunction, hepatosplenomegaly, clotting disorder, hyperferritinemia, pancytopenia, and high fever (66). Even though there is no published article explaining the role of macrophage polarization in SLE-MAS, hematologic and organ alterations in MAS are believed to result from the uncontrolled release of pro-inflammatory cytokines by abnormally activated T lymphocytes and macrophages (67). Given this implication of macrophages in the development of MAS and its association with SLE, it is reasonable that we consider this syndrome to be a part of this review.

### 4.2 Predisposing Factors of SLE-MAS

Although the etiology of SLE-MAS remains a mystery, several factors have been reported to trigger this syndrome. The most common ones are infection and SLE flare. Other reported factors include abortion and parturition (68, 69). While some articles have described that change of therapy and malignancy could trigger SLE-MAS (70), another study suggests that no link was found between these two factors and the development of SLE-MAS (69). Among these triggering factors, infection is the one that can probably explain the implication of macrophages in the development of SLE-MAS. When infection cannot be eliminated, the immune system becomes inappropriately stimulated, leading to hyperinflammation *via* an uncontrolled release of cytokines from immune cells such as macrophages. The resulting cytokine storm is believed to be responsible for the development of MAS (71). So far, The most reported infective triggers include Epstein-Barr virus (EBV) and cytomegalovirus (CMV) (72).

### 4.3 Pathogenesis of SLE-MAS

**Figure 2:**
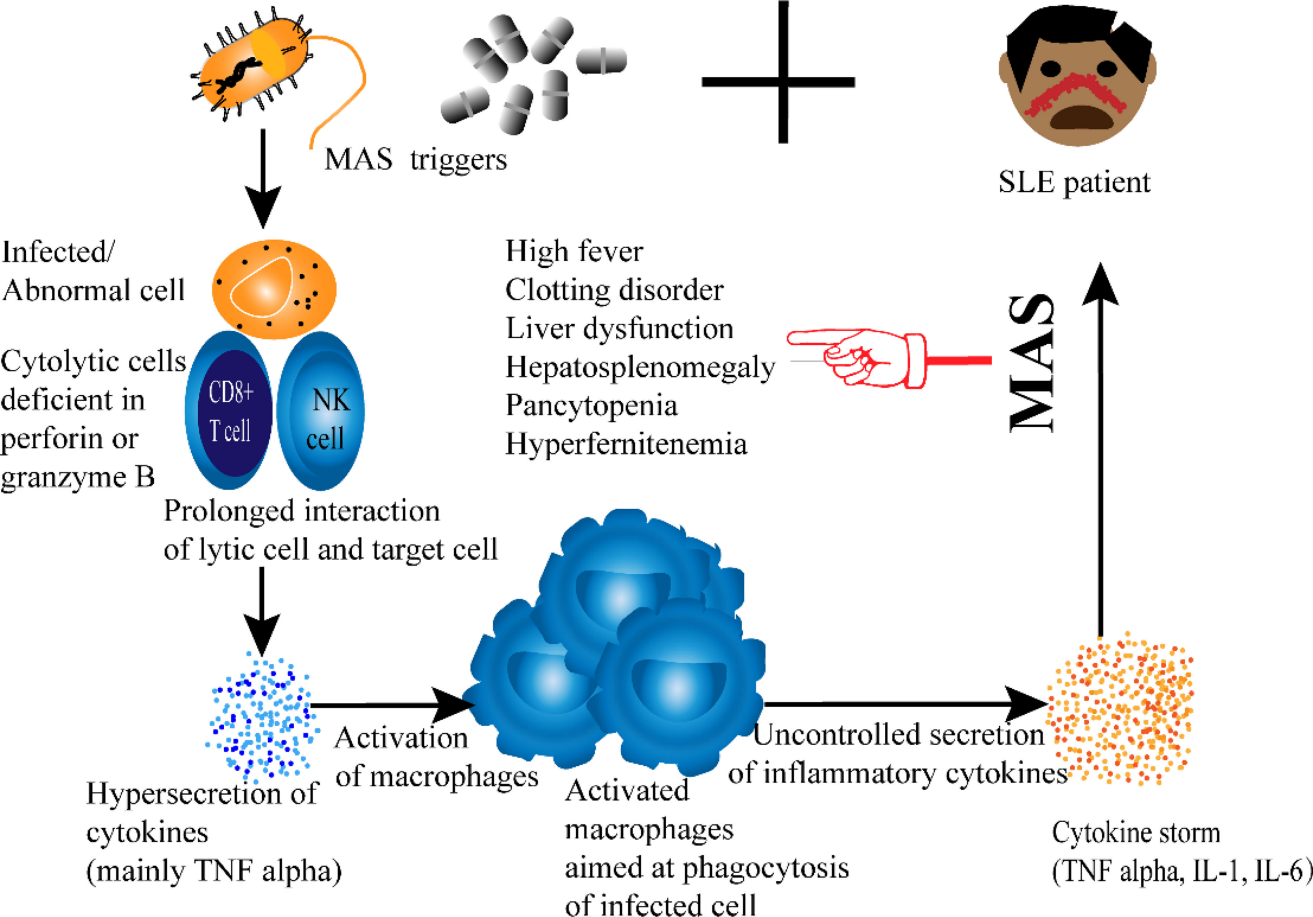
Proposed Pathogenesis of SLE-associated MAS.

Until now, the exact mechanism that leads to the development of MAS in SLE patients is not well established. Nevertheless, some pathways are believed to contribute to the pathogenesis of SLE-MAS. Exaggerated hypercytokinemia due to dysregulation of the macrophage-lymphocyte interaction is one way to understand the occurrence of SLE-MAS (71). **Figure 2**. Among cytokines, TNF-α is of particular significance as it is characteristic of SLE-MAS rather than other inflammatory diseases (71, 73). The importance of tumor necrosis factor is also reported in a recent study comparing cytokine levels in SLE patients with and without MAS. The same study has additionally shown that CXCL9 is likewise significantly elevated in SLE-MAS (74).

The mechanism of SLE-MAS could also be explained by IgM type antilymphocyte antibody (ALAB) and a mutation on the MEFV gene. The Presence of ALAB on lymphocytes is reported to be proportional to disease severity. In fact, in a young patient diagnosed with SLE-MAS, IgM-ALAB could be observed on the surface of lymphocytes during the acute phase but not with disease improvement (71). Even if we still do not understand the exact mechanism of the syndrome, this indicates a strong association between ALAB and SLE-MAS development. Validating this theory with a large number of patients would be beneficial since ALAB could be used to assess the activity of the disease.

Similarly, a retrospective study has demonstrated that serum sCD163 level is proportional to SLE-MAS activity (75). While these data suggest the possible usefulness of CD163 as a significant biomarker for SLE-MAS, further investigation is necessary to address the limitation of the current study. Apart from being retrospective, some patients in this study were treated with dexamethasone before sample collection. Since corticosteroids are known to upregulate the expression of CD163, this could have affected the serum sCD163 levels of some subjects.

In a nutshell, despite progress in clarifying the occurrence of SLE-MAS, studies on this syndrome are mostly conducted in a very limited number of patients. Possibly due to the rareness of MAS. This limitation emphasizes the need for extended investigations, especially since the syndrome can be fatal.

## 5 Treatment

The use of corticosteroids and immunosuppressants has considerably improved the prognosis of SLE (76, 77). However, despite being the cornerstones treatment for SLE, these drugs are associated with unwanted side effects, especially in children (78). Such issues bring up the great need for specific agents with fewer side effects. In order to reduce corticosteroids doses, several biological agents such as rituximab, belimumab have been developed, and some have yielded favorable results. Among the commonly used SLE medications, some are reported to impact macrophage polarization and therefore are summarized in **Table 2**.

**Table 2:** Current SLE medications with an impact on macrophage polarization.

Class of therapy	Mechanism of action	Impact on macrophages	Side effects
**Systemic therapy**			
**Corticosteroids**	Inhibit arachidonic acid and IL-1 formation and thus produce both anti-inflammatory and immunosuppressive effect	Change macrophage phenotype toward M2 polarization (79)	Infection, hypertension, glaucoma, osteoporosis, avascular necrosis, hyperglycemia, myopathy, weight gain (86, 87)
**Immunosuppressants**	**cyclosporin A**,		
**Tacrolimus** **IVIG**	Acts on T cells *via* calcineurin Acts on T cells *via* calcineurin Act on T and B cells, on interferon signaling pathway, and on defective elimination of immune complexes and other cellular debris	Promote M1 to M2 macrophage phenotype switch when combined to mesenchymal stem cells (80). Protect macrophages from LPS/INFγ-mediated apoptosis (81). Favors M2-like phenotype (82, 83) promotes the inflammatory actions of M2-like macrophages and reduces the proinflammatory activities of M1-like macrophages (84, 85)	Nephrotoxicity, cosmetic side effects (hypertrichosis, gingival hyperplasia) (88, 89) Nephrotoxicity, Neurotoxicity, diabetogenic, hypertension (90) Limited data, few reported adverse effects and mostly they are mild and transient. Severe reported adverse effects include Thromboembolic events and renal toxicity (91–94)

Many articles discuss the treatment options of systemic lupus erythematosus. However, to remain true to our topic, the treatment section of this review mainly focuses on two aspects. On the one hand, we discuss the management of patients with SLE-associated MAS, and on the other hand, we review the reported therapeutic options that target M1- and M2- like macrophages.

### 5.1 Treatment of SLE-MAS

For rheumatic diseases induced-MAS such as SLE-MAS, therapy is directed toward controlling the hyperinflammation state. Usually, this can be achieved through a combination of high-dose corticosteroids and immunosuppressants (95–97). Interestingly, hydroxychloroquine has been reported to decrease the probability of developing MAS in SLE patients. The mechanism is not fully understood, but it is hypothesized that hydroxychloroquine decreases the production of IL-1, IL-6, and TNF-α, which inhibits toll-like receptor (TLR) 3 and 7 and consequently lowers the likelihood of developing SLE-MAS (98).

Among immunosuppressants, some agents are reported to be more effective than others. Indeed, even if cyclosporin is a good immunosuppressant (77, 99), for maintenance of remission, the combination of tacrolimus and corticosteroids has been reported to provide a better outcome. In the same study, intravenous immunoglobulin (IVIG) is proposed to be more effective when combined with oral tacrolimus as compared to cyclosporin A (100). Reported biological agents that have been used to treat SLE-MAS include Rituximab (101) and the IL-1 receptor antagonist anakinra (96).

### 5.2 M1-and M2-Like Macrophages-Centered Therapies

#### 5.2.1 Therapeutic Approaches Affecting Both M1-and M2-Like Macrophages

Several *ex vivo, in vivo*, and *in vitro* studies have reported various experimental therapeutic approaches that affect the expression of M1-and M2-like macrophages. One of the investigated compounds is the Aryl hydrocarbon receptor (AhR) agonist indole-3-carbinol (I3C). AhR is a cytoplasmic receptor that exhibits numerous physiological functions in immune cells such as B cells, T cells, neutrophils, and macrophages (102). Emerging evidence suggests that I3C can be targeted to ameliorate diverse inflammatory and autoimmune conditions (103–106). Furthermore, it is reported that I3C can immunoregulate macrophages by activating AhR (107). An *ex vivo* study in SLE patients demonstrated that AhR activation could be mediated by I3C, resulting in a downregulation of M1 markers (CD86) and overexpression of M2 markers (CD163) (46). Meanwhile, an increase of anti-inflammatory cytokines and a decrease of inflammatory cytokines were induced, and consequently, inflammation was controlled (46). However, this study is not without limitations. Firstly, because CD163 can also be expressed by other cells and secondly because CD86 can be expressed by M2b macrophages (40, 108). Hence, the assessment of macrophages cannot be accurately achieved with these biomarkers alone. Similar *ex vivo* investigations demonstrated that I3C could assuredly alleviate lupus flares *via* macrophages regulation (109). The researchers have considered the environment’s influence on macrophage plasticity and used autologous plasma instead of M-CSF and GM-CSF synthetic growth factors so that *in vivo* conditions could be mimicked and bias avoided. Unfortunately, the small number of patients in this study and the characterization of M2-like macrophages using CD163+ minimizes its power.

Consistent with this theory, it is also suggested that sodium valproate alleviates inflammation in SLE patients. In fact, in an *ex vivo* study, sodium valproate was seen to upregulate the expression of anti-inflammatory cytokines from M2 phenotype macrophages (CD163) and to downregulate the expression of pro-inflammatory cytokines from M1 phenotype macrophages (CD86) (110). Nevertheless, the use of sodium valproate in neuropsychiatric lupus needs to be cautiously evaluated because the drug is reported to induce hyperammonemia which leads to a metabolic brain insult and the worsening of neuropsychiatric symptoms (111). In addition, previous studies have suggested a possible link between this drug and SLE development (112, 113). Such conflicting findings make the use of valproate in SLE controversial, and future studies need to look into this contradiction. Moreover, it is important to highlight again that CD163 alone cannot be used to characterize macrophages.

HMGB1 is another investigated molecule. As mentioned above, HMGB1 can trigger inflammation *via* inflammatory macrophages. However, an *ex vivo* study has revealed that the cooperation of C1q with HMGB1 can suppress inflammation by stimulating the polarization of M2-like macrophages (114). Such a finding is undoubtedly promising, but it should be noted that the study was conducted on murine lupus, and it is still unclear whether the same effect can be seen in human beings. Various preclinical studies have also explored the possible therapeutic effects of several inhibitors of HMGB1 (115) as well as HMGB1-specific antagonists (116), and the results are favorable.

Tuberous sclerosis complex 1 (TSC1) is an additional regulator of macrophage polarization. An *in vivo* study proved that the TSC1/2 complex helps to maintain macrophage homeostasis by regulating macrophage polarization through different signaling pathways (117). *Via* the Ras GTPase pathway, the TSC1/2 complex inhibits the M1 phenotype and contributes to autoimmune diseases’ amelioration. On the other hand, inhibition of the mammalian target of rapamycin (mTOR) pathway by TSC1/2 complex enhances M2 polarization (117). This makes TSC1 a potential regulator of macrophage polarization, and identifying its master role in macrophage activation could provide therapeutic strategies for autoimmune diseases. While the current study has demonstrated that TSC1/2 could prevent autoimmune diseases by controlling macrophage polarization, it does not address any specific autoimmune disease. Thus, validation in SLE needs to be determined.

Another compound that has been experimentally used to control SLE is pioglitazone. Pioglitazone corresponds to a peroxisome proliferator-activated receptor (PPAR)-γ agonist that is widely used to treat diabetes. In addition to its anti-diabetic effect, this drug also has anti-inflammatory properties (118). Such anti-inflammatory effect has been reported to benefit inflammatory diseases, including SLE. Indeed, an *ex vivo* study of newly diagnosed lupus patients showed that activation of PPAR-γ by pioglitazone could induce anti-inflammatory properties by promoting the expression of M2-like macrophages and decreasing the expression of M1-like macrophage (119). Likewise, an *in vivo* study has acknowledged this contribution of pioglitazone in alleviating murine LN. Even though the study has not mentioned macrophages’ involvement, it confirmed that the use of pioglitazone induces a protective effect of PPAR-γ against LN (120).

Clinically, Virgin olive oil (VOO) has been successfully used to ease symptoms in patients with lupus. A study conducted in humans proved that the phenolic fraction of extra virgin oil provided a beneficial effect on immune-inflammatory diseases such as SLE (121). Although this particular article does not state that this immunoregulation is achieved by macrophages, a more recent study has revealed that VOO dampens TLR4, which plays a role in both macrophage activation and polarization (122). Moreover, a comparison of the use of virgin olive oil and sunflower oil in murine lupus showed that the former could block the expression of M1 subtype macrophage while enhancing the M2 phenotype (122). Despite accumulated evidence showing the role of VOO in alleviating chronic immune-mediated diseases such as SLE, the exact mechanism is still unclear, and this limits its clinical use.

#### 5.2.2 Therapeutic Approaches Affecting M2-Like Macrophages

One of the processes that determine cell fate and cellular development is Notch signaling. Four Notch receptors (Notch1-4) have been identified in mammals, and impaired regulation of Notch1 signaling is associated with many diseases (123). The association of Notch signaling with SLE is not a new concept as a defective expression of Notch 1 in SLE T cells has been reported previously (124, 125). However, the involvement of Notch 1 in SLE macrophages has been newly established. Indeed, both *in vitro* and *in vivo* studies demonstrated that blockage of Notch 1 signaling could alleviate murine lupus by blunting M2b polarization (126). Although blocking Notch 1 could also suppress M1-like macrophage polarization and attenuate inflammation in cardiovascular diseases (127), data about Notch 1 signaling and M1 polarization in SLE is lacking. Further studies are required to explore this possible connection. Another investigated approach that could also improve SLE is the administration of PAM3CSK4 (PAM3), a TLR 2/1 agonist. By using PAM3, researchers were able to stimulate the polarization of monocytes from SLE patients toward M2-like macrophages *in vitro*. Moreover, PAM3 treatment delayed the disease progression and prolonged survival in murine models (128, 129). Similar to the inducers of M2 polarization mentioned above, the use of artemisinin *in vivo* has offered a therapeutic effect on female mice with lupus by stimulating the secretion of anti-inflammatory cytokines from M2-like macrophages (130). Another compound that could help patients with lupus is Liver x-receptor Alpha (LXRα). LXRα is one of the two isoforms of liver X receptors, a nuclear receptor superfamily member. Besides being an essential receptor for cholesterol regulation, this protein also regulates the activation of macrophages, and its therapeutic effects on autoimmune diseases are gaining much attention (131, 132). Recently, an *in vivo* study has shown that LXR α could promote M2-like macrophages polarization in murine lupus and subsequently prevent the occurrence of diffuse alveolar hemorrhage, a deadly complication of lupus (133).

Clinically, azithromycin, a macrolide antibiotic useful for controlling inflammation in various diseases (134), has been proven to ease lupus flare *via* the regulation of M2-like macrophages. *In vivo* and *in vitro* studies in SLE patients revealed that azithromycin promotes M2 polarization *via* the pl3k/Akt pathway, resulting in the suppression of inflammation and SLE remission (135). Albeit its reported side effects (136), azithromycin does have a good record of safety, and exploring it could offer a new therapeutic strategy for lupus patients. Another drug that favors the M2 phenotype is steroid. A recent study has demonstrated that the clinical use of steroids can change macrophage phenotype toward M2 polarization and consequently suppress inflammation in lupus patients (79). However, this phenotype switch is also reported to worsen LN. The study showed that by promoting M2 polarization, steroids also induce interstitial fibrosis and exacerbate chronic glomerular lesions (79). This finding highlights the great need to evaluate the possible side effects of inducing a macrophage phenotype change.

## 6 Outstanding Gaps and Future Direction

The appreciation of macrophage polarization and plasticity in SLE has allowed scientists to gain a better insight into the development of this autoimmune disease. Even though it is still yet to be determined whether the deregulation of macrophages phenotypes observed in SLE is a cause or consequence of the pathogenic process, numerous studies have reported an amelioration of the disease activity after restoring the balance between M1/M2 phenotypes. Indeed, exposure of M1-like macrophages to M2 modulators or vice versa was seen to induce re-polarization of macrophages and consequently control lupus flares both *in vitro* and *in vivo*. Currently, various new and exciting macrophages-based therapeutic strategies are being exploited, and they appear to be promising. These therapeutic approaches bring a glimmer of hope for the lupus community as they provide us with a new pathway toward the treatment of SLE. However, there is still work to be done, and several gaps need to be addressed in the future in order to get more clarification about these macrophages-centered therapies. First of all, scientists have established the effect of various compounds on macrophages polarization, but their possible physiological effects on other cells are poorly studied. SLE is a systemic disease with numerous manifestations in different organs. While inducing a macrophage phenotype switch has been proven to ameliorate some aspects of lupus flares, it could also worsen other manifestations of the disease, as it is seen with corticosteroids (79). Such possible outcome needs to be carefully evaluated and enlightened in future work. Secondly, the influence exerted by the environment on the plasticity of macrophages is non-negligible, and this needs to be taken into consideration. *In vitro* findings cannot directly be translated into *in vivo*. Therefore, we still do not know how some of these macrophages-centered therapies would behave in the human body. Paying closer attention to this fact is necessary in order to exploit these immune cells better. Finally, the field could also benefit from more studies in children because even though they do not represent the majority of the SLE community, they do have the most severe manifestations, and children are known to function differently from adults.

## Author Contributions

YJ was resident and provided the main plan and conceptual ideas. MA was resident and wrote the manuscript. XW was professor and supervised the work. All authors contributed to the article and approved the submitted version.

## Funding

This work was supported by the Natural Science Foundation of Hunan Province (No.2021JJ30960) and Natural Science Foundation of Hunan Province (No.2021JJ40836).

## Conflict of Interest

The authors declare that the research was conducted in the absence of any commercial or financial relationships that could be construed as a potential conflict of interest.

## Publisher’s Note

All claims expressed in this article are solely those of the authors and do not necessarily represent those of their affiliated organizations, or those of the publisher, the editors and the reviewers. Any product that may be evaluated in this article, or claim that may be made by its manufacturer, is not guaranteed or endorsed by the publisher.
